# Do We Need to Monitor for Graves’ Disease After a Diagnosis of Pemphigoid Gestationis?

**DOI:** 10.7759/cureus.48972

**Published:** 2023-11-17

**Authors:** Katherine C Awh, Sylvia Hsu

**Affiliations:** 1 Dermatology, Thomas Jefferson University, Philadelphia, USA; 2 Dermatology, Temple University Hospital, Philadelphia, USA

**Keywords:** thyroid disorder, pemphigoid, autoimmune bullous disease, hyperthyroidism, graves’ disease, pemphigoid gestationis

## Abstract

Pemphigoid gestationis (PG) is a rare autoimmune bullous disease that occurs during pregnancy or the postpartum period. PG has been associated with an increased risk of Graves' disease possibly due to shared genetic factors and immune system fluctuations during pregnancy. However, the evidence supporting the association between PG and Graves' disease is mixed. Although dermatologists are cautioned to watch for Graves’ disease in patients with a history of PG, this guidance is based on a single cohort where most patients were diagnosed with Graves' disease prior to PG onset. Recent data failed to find an association between Graves’ disease and PG but did not capture the lifetime risk of Graves’ disease in these patients. Future studies could focus on long-term follow-up of females with PG, shedding light on the lifetime risk profiles of these patients.

## Editorial

Pemphigoid gestationis (PG) is a rare autoimmune subepidermal bullous disease that is characterized by the eruption of pruritic bullae during pregnancy and the postpartum period [1]. The eruption typically begins on the periumbilical abdomen and spreads to the limbs and trunk, sparing the face and mucous membranes [1]. PG usually develops in the second trimester of pregnancy and resolves spontaneously after delivery, recurring with an earlier onset in each subsequent pregnancy [1]. The exact etiology of PG is unknown, but it is hypothesized to be related to a pregnancy-induced immune response in which autoantibodies target the hemidesmosomal protein BP180 (BPAG2), also known as type XVII collagen [2]. While there are no established guidelines for treating PG, systemic and topical corticosteroids have been the mainstay of treatment. Dermatologists are commonly taught that 10% of females with PG develop Graves’ disease, requiring careful monitoring for Graves’ disease in these patients [1]. However, this 10% statistic originates from a singular cohort of 87 patients with PG, nine (10.3%) of whom had a diagnosis of Graves’ disease at some point [3]. Notably, over half of these patients were already diagnosed with Graves’ disease prior to developing PG, necessitating a closer look at monitoring conventions [4].

The mechanism underlying the association between PG and Graves' disease is not well understood and is likely due to a shared genetic predisposition. Major histocompatibility complex (MHC) class II human leukocyte antigen (HLA) antigen DR3 is implicated in both diseases [4]. It has also been suggested that pregnancy-related hormonal changes and fluctuations in the immune response may contribute to the development of both conditions [5]. A shift towards a T helper (Th) 2 immune response has been indicated as a possible trigger of autoimmune bullous diseases during pregnancy, and Graves' disease is often seen as a Th2-dominated disease [6,7]. Nevertheless, there is no consistent pattern concerning the onset of Graves’ disease in relation to PG. Cases of Graves’ disease have been noted prior to the first PG-associated pregnancy, during the PG-associated pregnancy, or up to a dozen years post-partum [4,8,9].

Unfortunately, our understanding of the long-term risks of autoimmune diseases in PG patients is presently limited, and available studies are limited to several case reports and two cohort studies [3,9,10]. To the best of our knowledge, the largest study of PG and Graves’ disease to date is a recent population-based case-control study from Germany [10]. The authors found no association between PG and hyperthyroidism. Hyperthyroidism was present in 5/126 (4.0%) of PG cases compared to 39/933 (4.2%) of controls [10]. However, this cross-sectional study measured comorbid hyperthyroidism in cases of PG between 2008 and 2011 and did not capture the lifetime risk of Graves’ disease in these patients. Across the remaining studies, there is a similar dearth of long-term data due to the focus on current or recent cases of PG with limited follow-up. Without long-term data on the risk of autoimmune diseases in these patients, we cannot rule out an association between Graves’ disease and PG.

In conclusion, the evidence supporting the association between PG and Graves' disease is mixed. Potential etiologies include shared genetic factors and immune system fluctuations during pregnancy. Although dermatologists are cautioned to watch for Graves’ disease in patients with a history of PG, this guidance is based on a single cohort where most patients were diagnosed with Graves' disease prior to PG onset. The largest study to date failed to find an association between Graves’ disease and PG; however, this did not capture the lifetime risk of Graves’ disease in these patients. Future studies could focus on long-term follow-up of females with PG, shedding light on the lifetime risk profiles of these patients.

## References

[1] Lipozenčić J, Ljubojevic S, Bukvić-Mokos Z (2012). Pemphigoid gestationis. Clin Dermatol.

[2] Semkova K, Black M (2009). Pemphigoid gestationis: current insights into pathogenesis and treatment. Eur J Obstet Gynecol Reprod Biol.

[3] Jenkins RE, Hern S, Black MM (1999). Clinical features and management of 87 patients with pemphigoid gestationis. Clin Exp Dermatol.

[4] Shornick JK, Black MM (1992). Secondary autoimmune diseases in herpes gestationis (pemphigoid gestationis). J Am Acad Dermatol.

[5] Genovese G, Derlino F, Berti E, Marzano AV (2020). Treatment of autoimmune bullous diseases during pregnancy and lactation: a review focusing on pemphigus and pemphigoid gestationis. Front Pharmacol.

[6] Nagayama Y, Mizuguchi H, Hayakawa T, Niwa M, McLachlan SM, Rapoport B (2003). Prevention of autoantibody-mediated Graves'-like hyperthyroidism in mice with IL-4, a Th2 cytokine. J Immunol.

[7] Feliciani C, Genovese G, D'astolto R, Pontini P, Marzano AV (2019). Autoimmune bullous diseases during pregnancy: insight into pathogenetic mechanisms and clinical features. G Ital Dermatol Venereol.

[8] Holmes RC, Black MM (1980). Herpes gestationis: a possible association with autoimmune thyrotoxicosis (Graves' disease). J Am Acad Dermatol.

[9] Hon KL, Chiu LS, Lam MC, Choi CL, Chan S, Luk NM (2007). Normal neonatal outcome in a Chinese woman with pemphigoid gestationis, Graves' disease, and history of placental chorioangioma. Int J Dermatol.

[10] Kridin K, Hübner F, Linder R, Schmidt E (2022). The association of six autoimmune bullous diseases with thyroid disorders: a population-based study. J Eur Acad Dermatol Venereol.

